# Epidermal growth factor enhances osteogenic differentiation of dental pulp stem cells *in vitro*

**DOI:** 10.1186/s13005-015-0086-5

**Published:** 2015-09-03

**Authors:** Casiano Del Angel-Mosqueda, Yolanda Gutiérrez-Puente, Ada Pricila López-Lozano, Ricardo Emmanuel Romero-Zavaleta, Andrés Mendiola-Jiménez, Carlos Eduardo Medina-De la Garza, Marcela Márquez-M, Myriam Angélica De la Garza-Ramos

**Affiliations:** Unidad de Odontología Integral y Especialidades, Centro de Investigación y Desarrollo en Ciencias de la Salud, Universidad Autónoma de Nuevo León, Monterrey Nuevo León, México; Instituto de Biotecnología, Facultad de Ciencias Biológicas, Universidad Autónoma de Nuevo León, San Nicolás de los Garza, Nuevo León, México; Departamento de Química, Facultad de Ciencias Biológicas, Universidad Autónoma de Nuevo León, San Nicolás de los Garza, Nuevo León, México; Facultad de Odontología, Universidad Autónoma de Nuevo León, Monterrey Nuevo León, México; Facultad de Medicina, Universidad Autónoma de Nuevo León, Monterrey Nuevo León, México; Department of Oncology-Pathology, CCK, Karolinska Institutet, Stockholm, Sweden

**Keywords:** Dental pulp stem cells, Epidermal growth factor, Basic fibroblast growth factor, Osteogenic differentiation, Bone mineralization, Bone remodelling

## Abstract

**Introduction:**

Epidermal growth factor (EGF) and basic fibroblast growth factor (bFGF) play an important role in extracellular matrix mineralization, a complex process required for proper bone regeneration, one of the biggest challenges in dentistry. The purpose of this study was to evaluate the osteogenic potential of EGF and bFGF on dental pulp stem cells (DPSCs).

**Material and methods:**

Human DPSCs were isolated using CD105 magnetic microbeads and characterized by flow cytometry. To induce osteoblast differentiation, the cells were cultured in osteogenic medium supplemented with EGF or bFGF at a low concentration. Cell morphology and expression of CD146 and CD10 surface markers were analyzed using fluorescence microscopy. To measure mineralization, an alizarin red S assay was performed and typical markers of osteoblastic phenotype were evaluated by RT-PCR.

**Results:**

EGF treatment induced morphological changes and suppression of CD146 and CD10 markers. Additionally, the cells were capable of producing calcium deposits and increasing the mRNA expression to alkaline phosphatase (ALP) and osteocalcin (OCN) in relation to control groups (*p* < 0.001). However, bFGF treatment showed an inhibitory effect.

**Conclusion:**

These data suggests that DPSCs in combination with EGF could be an effective stem cell-based therapy for bone tissue engineering applications in periodontics and oral implantology.

## Introduction

The multi-lineage differentiation capacity of mesenchymal stem cells (MSCs) has been amply studied in recent years because of its implication in tissue engineering and regenerative medicine [1, 2]; however, this field is currently faced with the critical challenge of developing novel approaches to regenerate large bone defects. Some years ago, Gronthos and colleagues isolated dental pulp stem cells (DPSCs) from human third molars confirming that these cells present the ability to differentiate into odontogenic/osteogenic cells [3–5]. Previous reports have shown that the osteogenic differentiation on DPSCs is successfully induced by chemical cues such as dexamethasone, ascorbic acid, and β-glycerophosphate [6–8]. Although these compounds have proven efficacy, analysis of the role of growth factors in osteogenesis has been the aim of several studies focused on improving extracellular matrix mineralization, a physiological process characterized by high expression of alkaline phosphatase (ALP) and osteocalcin (OCN), followed by calcium deposition [9, 10].

Epidermal growth factor (EGF) and basic fibroblast growth factor (bFGF) are powerful mitogens for many cell types including MSCs [11–13]. Ideally, it is expected that these factors maintain the self-renewal and multi-potency capacities of these cells [14] but it is known that they can also promote differentiation towards specialized lineages such as osteoblasts, a process largely controlled by various growth factors [15, 16]. Certain studies show that bFGF affects osteogenic differentiation of DPSCs [17] through inhibition of ALP enzymatic activity and mineralization [18]. This effect has also been shown in stem cells from human exfoliated deciduous teeth (SHED) and periodontal ligament stem cells (PDLSCs) [19, 20]. On the other hand, it is well-known that an extensive variety of mesenchymal cells normally express the epidermal growth factor receptor (EGFR), a tyrosine kinase receptor that activates intracellular signalling pathways that determine their fate [21–23]. Emerging evidence suggests that EGF works as an enhancer of mineralization during differentiation of MSCs derived from bone marrow [24, 25]; however, the effect of EGF on osteogenic differentiation of DPSCs is unknown.

The purpose of this study was to evaluate the role of EGF and bFGF in order to identify crucial growth factors associated with enhancing osteogenic differentiation of DPSCs. We hypothesized that EGF supplementation may increase mineralization on the osteogenic differentiation of these cells. Our results provide evidence that EGF treatment, but not bFGF, is capable of increasing calcium deposit formation as well as ALP and OCN gene expression compared to traditional osteogenic medium. These observations indicate that EGF could be an effective adjuvant for improving bone regeneration in periodontics and oral implantology.

## Material and methods

### Subjects

Pulp samples were obtained from 12 human premolars extracted for orthodontic purposes from healthy patients; finally, the dental pulp tissues of the youngest patient (18 years of age) were used. The protocol was approved by the Ethics Committee, School of Dentistry of the Universidad Autónoma de Nuevo León (UANL) and performed in accordance with the ethical standards laid down in the 1964 Declaration of Helsinki. Informed consent was obtained from all donors.

### Cell culture

Dental pulp explants were digested with 3 mg/ml collagenase type I and 4 mg/ml dispase (Sigma-Aldrich, St. Louis, MO, USA) at 37 °C for 1 h. The cell suspension was centrifuged at 300 g for 10 min, washed and then filtered through a 70 μm nylon filter (BD Biosciences, San Jose, CA, USA). Dental pulp cells were maintained in α-modified Eagle's medium (α-MEM) supplemented with 10 % fetal bovine serum (FBS) (Gibco-Invitrogen, Carlsbad, CA, USA), 2 mM L-glutamine, 100 U/ml penicillin, 100 μg/ml streptomycin and 0.25 μg/ml amphotericin B (Sigma-Aldrich) at 37 °C in a humidified atmosphere with 5 % CO_2_ for 3 weeks. The medium was renewed every 3 days.

### Magnetic cell sorting

Cell isolation was performed following the manufacturer’s protocol. Briefly, cultured cells were resuspended in PBS with 1 % bovine serum albumin (BSA) (Sigma-Aldrich) and then incubated with CD105 magnetic microbeads (Miltenyi Biotech, Bergish Gladbach, Germany) for 15 min at 4 °C. Cells were washed and loaded into a MS column placed in the magnetic field of a MiniMACS™ Separator (Miltenyi Biotech). Magnetically-labelled cells were collected and subcultured until passage 3 under the same growth conditions.

### Flow cytometry analysis

To confirm the typical MSC immunophenotype, magnetic-isolated cells were incubated with the following monoclonal antibodies: CD105-FITC, CD73-PE, CD13-PE, CD45-FITC, CD34-PE, HLA-DR-PerCp, CD14-PE, CD11b-PE (BD Biosciences) and CD90-FITC (Miltenyi Biotech). Antibodies were added to ~1 x 10^5^ cells per sample and then incubated for 30 min at 4 °C in dark. Stained cells were washed and then resuspended in PBS with 4 % paraformaldehyde. All samples were analyzed in a FACSCalibur™ flow cytometer system (BD Biosciences).

### Formalin-induced fluorescence assay

DPSCs were plated onto 6-well plates (Corning-Costar, Corning, NY, USA) at a density of ~3 x 10^4^ per well and cultured for 7 days in α-MEM as a negative control, and osteogenic medium (OM) as a positive control, composed of α-MEM, 10^−7^ M dexamethasone, 50 μg/ml ascorbic acid and 10 mM β-glycerophosphate (Sigma-Aldrich). At the same time, cells were incubated with OM containing 10 ng/ml of human EGF (OM + EGF) (Miltenyi Biotech) and OM containing 10 ng/ml of human bFGF (OM + bFGF) (Life Technologies, Rockville, MD, USA). Cultured cells were washed and then fixed with 10 % neutral-buffered formalin (BDH Chemicals, Ltd, UK) for 30 min. Fixed cells were incubated with 1 μg/ml DAPI (Thermo Scientific, Waltham, MA, USA) at room temperature for 5 min in dark. Cells were analyzed in a Zeiss Axiovert 200 M fluorescence microscope (Carl Zeiss, Göttingen, Germany).

### Immunocytochemistry

DPSCs were plated onto 8-well chamber slides (Lab-Tek Chamber Slide, Nunc, Germany) at a density ~2.5 x 10^3^ per well and maintained in α-MEM, OM, OM + EGF and OM + bFGF for 7 days. Cultured cells were fixed with cold methanol for 10 min and then incubated in PBS with 2 % BSA at room temperature for 30 min. Fixed cells were incubated with mouse anti-human CD146-FITC (Miltenyi Biotech) and mouse anti-human CD10-FITC (BD Biosciences) monoclonal antibodies, counterstained with DAPI and then analyzed by fluorescence microscopy.

### Osteogenic differentiation

DPSCs were plated onto 24-well plates (Corning-Costar) at a density of ~6 x 10^3^ cells per well and cultivated in α-MEM for 24 h. The DPSCs were washed and then maintained in different culture media: α-MEM, OM, OM + EGF and OM + bFGF at 37 °C in a humidified atmosphere with 5 % CO_2_ for 21 days. All media were renewed every 3 days.

### Alizarin red S assay

After 21 days of osteogenic induction, the cells were fixed with 10 % neutral-buffered formalin for 30 min. Fixed cells were washed and then incubated with 2 % alizarin red S (ARS) (pH 4.2) (Sigma-Aldrich) at room temperature for 30 min in dark with gentle shaking. After staining, they were washed 4 times with PBS. The cells were analyzed by light microscopy and then incubated with cetylpyridinium chloride (CPC) 100 mM at 37 °C for 1 h to solubilize the extracellular calcium deposits attached to ARS. Two hundred microliters of each sample were transferred onto 96-well black plates (Corning-Costar). The ARS concentration was determined by absorbance at 495 nm in an iMark™ Absorbance Microplate Reader (Bio-Rad, Hercules, CA, USA) [26].

### Reverse transcriptase polymerase chain reaction (RT-PCR)

Total RNA from DPSCs, cultured in α-MEM, OM, OM + EGF and OM + bFGF was isolated using the TRIzol method (Invitrogen Corp, Carlsbad, CA, USA). For the cDNA synthesis, the ImProm-II Reverse Transcription System kit (Promega, Madison, WI, USA) was used according to the manufacturer’s instructions. PCR reactions to β-actin, alkaline phosphatase (ALP), bone sialoprotein (BSP), osteocalcin (OCN) and osteopontin (OPN) were performed in a MJ-Mini™ Staff Thermal cycler (Bio-Rad), following the protocol previously described [27]. PCR products were resolved on 1.5 % agarose gel electrophoresis, running at 100 V for 35 min. The gels were stained with 1 μg/ml ethidium bromide (Bio Basic Inc, Markham, ON, Canada) and displayed in a UV Transilluminator Doc™ Gel (Bio-Rad). All the reagents were used as a negative control for PCR except cDNA. In our study, all tests were performed three times (Table 1).

**Table 1:** Primer sequences for osteogenic differentiation analysis using reverse transcriptase-polymerase chain reaction (RT-PCR)

Gene	Sequence of oligonucleotides (5’- 3’)	Tm °C
β-Actin	Forward: GGCATCCTGACCCTGAAGTA Reverse: GGGGTGTTGAAGGTCTCAAA	51
OCN	Forward: GAGCCCCAGTCCCCTACC Reverse: CCGATAGAGGTCCTGAAAG	58
BSP	Forward: CAGCGGAGGAGACAATGGAG Reverse: TTCAACGGTGGTGGTTTTCC	58
OPN	Forward: CAACGAAAGCCATGACCACA Reverse: CAGGTCCGTGGGAAAATCAG	54
ALP	Forward: GGTGAACCGCAACTGGTACT Reverse: CCCACCTTGGCTGTAGTCAT	54

### Statistical analysis

The ARS levels were analyzed using one-way analysis of variance (ANOVA) and Tukey’s test for multiple comparisons among groups and *p*-values < 0.01 were considered statistically significant in all treatments. Data analysis was performed with SPSS software (SPSS Inc, Chicago, IL, USA).

## Results

### Isolation and phenotypic characterization of DPSCs

**Fig. 1:**
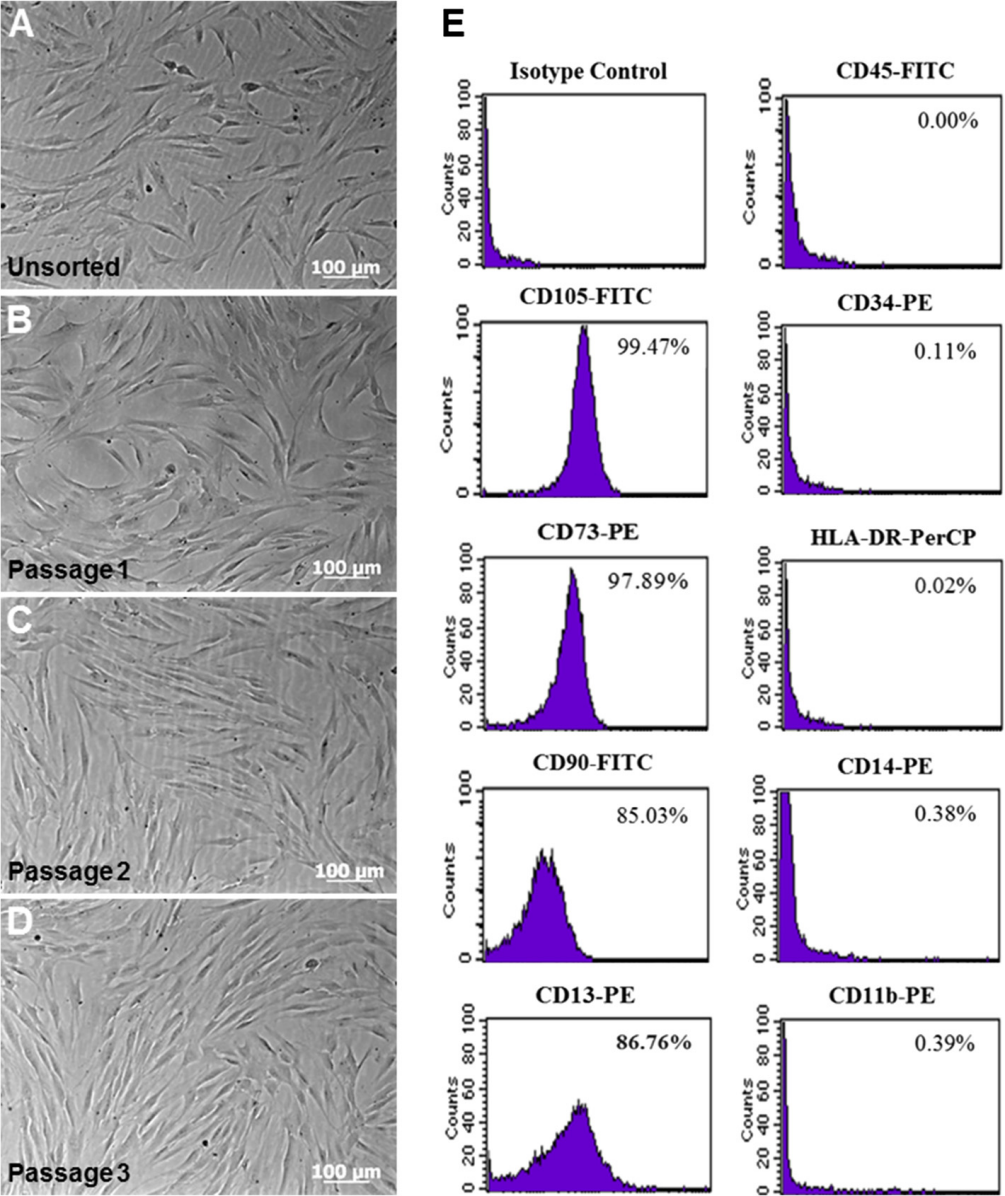
Cell culture and flow cytometry analysis of isolated dental pulp stem cells (DPSCs). **a** Representative phase-contrast micrographs shows unsorted-cells derived from human dental pulp tissue after 14 days of cell culture. **b**–**d** CD105^+^ magnetically-sorted DPSCs cultured in α-MEM without osteogenic induction. Morphologically, cells appear as typical fibroblastic and spindle shape during 3 passages. Original magnification 10x, scale bar =100 μm. **e** Flow cytometric analysis presented as histograms that show cell fluorescence intensity on the horizontal axis and cell frequency distribution on the vertical axis. Percentage results show positive expression to immunophenotype associated with mesenchymal stem cell (MSC) lineage as well as a lack of expression for hematopoietic markers

Adherent unsorted cells showed different sizes and morphologies after 3 weeks under cell growth conditions (Fig. 1a), in contrast, CD105^+^ magnetically-sorted cells showed a relatively homogeneous morphology characterized by spindle-shaped appearance with oval-central nuclei. Additionally, several colony-forming unit fibroblasts (CFU-F) were observed until passage 3 (Fig. 1b–d). Sorted cells had positive or negative expression by flow cytometry to the following surface markers: 99.47 % CD105-FITC, 97.89 % CD73-PE, 85.03 % CD90-FITC, 86.76 % CD13-PE, 0 % CD45-FITC, 0.11 % CD34-PE, 0.02 % HLA-DR-PerCp, 0.38 % CD14-PE and 0.39 % CD11b-PE (Fig. 1e). These results confirm that our cell culture presented the typical MSC immunophenotype: CD105^+^/CD73^+^/CD90^+^/CD13^+^/CD45^−^/CD34^−^/HLA-DR^−^/CD14^−^/CD11b^−^.

### Morphological changes and expression of CD146 and CD10 surface markers

**Fig. 2:**
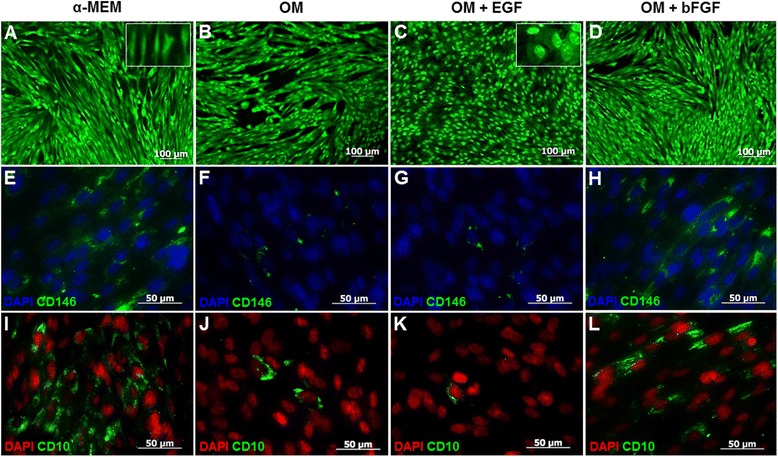
Morphological analysis and expression of mesenchymal stem cell (MSC) markers on dental pulp stem cells (DPSCs). **a**–**d** Morphological changes after 7 days of osteoblast differentiation. DPSCs begin to lose the typical spindle-shape MSC morphology and become osteoblast-like cells. Original magnification 10x, scale bar =100 μm. **e**–**h** Stemness biomarkers were analyzed by immunocytochemistry. Representative immunofluorescence images show changes in CD146 surface marker expression on DPSCs after osteogenic induction for 1 week. **i**–**l** Expression levels of the CD10 marker. Cells were stained with primary antibodies: mouse anti-human CD146-FITC, mouse anti-human CD10-FITC. Original magnification 40x, scale bar =50 μm

After 7 days in α-MEM incubation, DPSCs showed a fibroblastic-elongated morphology and tended to align themselves in parallel lines (Fig. 2a). Similar cell morphology was also observed in OM treated-cells (Fig. 2b). However, DPSCs in OM + EGF treatment showed clear morphological differences, characterized by polygonal-shaped appearance with spherical-peripheral nuclei and low cytoplasm content (Fig. 2c); moreover, these changes in OM + bFGF treatment were not observed (Fig. 2d). Additionally, the presence of EGF seems to induce a different organization pattern in cell culture, in comparison to the OM group. The highest cell confluence was observed in cells incubated with EGF or bFGF, in relation to α-MEM and OM control groups. Immunofluorescence analysis confirmed that cells cultivated in α-MEM for 7 days were highly positive to CD146 and CD10 surface markers (Fig. 2e, i). Although, the OM group was capable of decreasing expression of both markers (Fig. 2f, j), EGF treated-cells showed the strongest inhibitory effect (Fig. 2g, k). In contrast, bFGF treated-cells seem to maintain expression levels in relation to α-MEM group (Fig. 2h, l).

### Extracellular calcium deposition by ARS assay

**Fig. 3:**
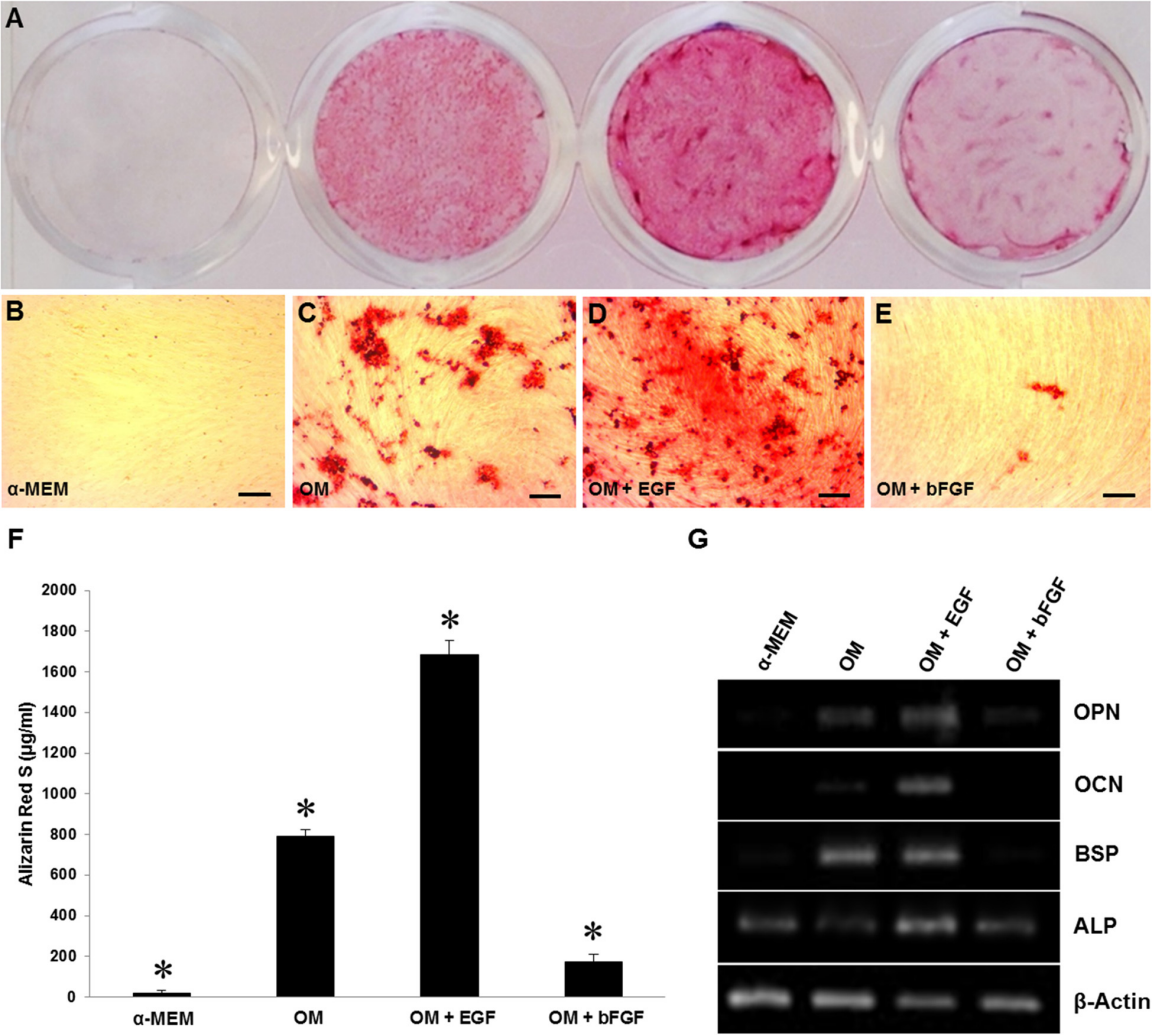
Mineralization and gene expression of osteoblast markers. **a**–**e** Cells were treated with α-MEM, OM, OM + EGF and OM + bFGF for 21 days and stained with alizarin red S (ARS), micrographs show extracellular calcium deposition. Original magnification 10x, scale bar =100 μm. **f** Calcified areas were quantified. Total calcium content was significantly increased with EGF treatment compared to all groups (*p* < 0.001). Error bars indicate mean ± SD (*n* = 3), asterisk indicate statistical significance (*p* < 0.001). **g** Total RNA was extracted from induced osteoblast-like cells. mRNA expression of the osteogenic markers, alkaline phosphatase (ALP), bone sialoprotein (BSP), osteocalcin (OCN) and osteopontin (OPN), was examined by RT-PCR. The housekeeping gene β-actin was used as a control for the PCR reaction. The results of this study confirm the participation of these genes in regulating the mineralization process of the extracellular matrix. All treatments were performed in triplicate

After 21 days under osteogenic induction, a complete cell confluence in all treatments was observed. At this stage, the α-MEM group was negative to ARS (18.81 μg/ml); however, in OM, OM + EGF, and OM + bFGF treatments, calcium deposition were observed (Fig. 3a). Microscopic analysis confirmed the absence of mineralized nodules in α-MEM (Fig. 3b). DPSCs treated only with OM showed high levels of ARS (792.64 μg/ml) and prominent mineralization nodules (Fig. 3c). Interestingly, OM supplemented with EGF induced a clear increase in abundance and size of calcium deposits (Fig. 3d), in addition to a significant increase in the mineralization levels evaluated by ARS (1686.31 μg/ml), in comparison to OM control group (Fig. 3f). In contrast, supplementation with bFGF showed a statistical difference with ARS (174.87 μg/ml) with respect to OM or OM + EGF, but the number of mineralized nodules were fewer than OM, suggesting an inhibitory effect (Fig. 3e).

### Gene expression by RT-PCR

After 21 days of cell culture, gene expression of OCN was negative in the α-MEM group. In addition, the OM group was positive for this osteoblast-phenotypic marker; however, its expression level was superior due to the presence of EGF in the culture medium, suggesting its importance during osteogenesis. Contrary to these effects, the addition of bFGF resulted in a decrease in BSP, OCN and OPN expression with respect to OM treated-cells (Fig. 3g).

## Discussion

Growth factors are recognized for their active participation in many biological processes such as cell migration, proliferation and differentiation [11, 28]. In the osteogenic context, it is also known that some of these factors play an essential role in bone regeneration since they are responsible for triggering cell specific signalling pathways that allow expression of bone morphogenetic proteins (BMPs), which are molecules centrally involved in extracellular matrix mineralization and damage bone repair [29–31].

Our results provide evidence that supplementation with EGF enhances osteogenic mineralization on DPSCs during cell differentiation, suggesting its important role in favoring this cell fate. EGF and bFGF supplementation is commonly used to ensure survival and proliferation of MSCs cultured under serum-free conditions [32–34]; however, recent studies suggest that EGF added to traditional osteogenic medium not only promotes cell proliferation but also enhances mineralization of MSCs derived from bone marrow [24, 25, 35]. We have found that DPSCs are an excellent alternative to use instead of bone marrow for cell therapy; however, a challenge to overcome is the small amount of dental pulp tissue obtained; it is for this reason that in our study the cells were obtained from human premolars extracted for orthodontic purposes.

It is known that growth factors such as IGF-1, TGF-β and TNF-α enhance osteogenic differentiation of DPSCs [36–38]. Additionally, a recent study showed that 12 or 24 h of EGF treatment enhanced chemokine IL-8 and BMP-2 expression in human periodontal ligament cells (HPDLCs) [39]. Since BMPs play a critical role in the mineralization process [40, 41], one can predict that the supernatant cell culture of EGF-treated cells could promote osteogenic differentiation more efficiently. Based on our findings, EGF can be used alone or in combination with any of these factors to achieve a synergistic effect. It is noteworthy that previous studies with EGF do not give similar results, but sometimes observations can be antagonistic. In this respect, some studies have reported an inhibitory effect induced of EGF on osteogenic differentiation of MSCs not derived from dental pulp [42, 43]. A possible explanation for these heterogeneous results could be variation of cell origin of MSCs used in each study. This strengthens the importance of characterizing MSCs derived from dental pulp. Another possible reason for this discrepancy is the use of primary or immortalized cells as well as their heterogeneity. In order to reduce this heterogeneity, our experiments were performed using magnetically-labelled DPSCs CD105^+^ thus favoring the phenotype of primary cells, which could be closer to an *in vivo* situation than the experiments done with immortalized cells.

On the other hand, we also observed that bFGF was not able to exert effects similar to EGF and was a significant inhibitory factor for mineralization and differentiation towards osteoblast-like cells. This confirms that not all growth factors related to the proliferation and expansion of DPSCs are capable of enhancing osteogenic mineralization. Similarly, these effects were also observed by Li et al. [17–19] on SHED, although they evaluated a higher bFGF concentration (100 ng/ml), which is 10 times more concentrated than that of our experiments.

Cell morphology has been used as an important indicator to characterize and assess cell quality [44, 45]; we observed that morphological changes can also be used to follow mesenchymal-osteoblast cell transition from DPSCs at early stages (1 week). Here we found typical osteoblast morphology in advanced stages of cell differentiation (3 weeks) associated with high levels of calcium deposits. During the odontogenic differentiation, it is known that there is an up-regulation of odontoblast-specific genes, including dentin sialophosphoprotein (DSPP) and dentin matrix protein 1 (DMP1) [46, 47]. In our study due to dental origin of the cells is possible an odontogenic differentiation too; these results suggest that cell morphology in early stages of cell differentiation can be an important complementary data to assess cell lineage; however, in a confluent cell culture it is technically complicated to measure those morphological changes. It is noteworthy that after 1 week in osteogenic conditions, the DPSCs changed their colony-cell distribution; moreover, a greater cell adherence can be observed. As a general consensus, some surface markers are included within the minimum criteria for defining MSCs [48]; however, others markers have been associated with MSC lineage, such as CD146 and CD10, both expressed on DPSCs [49, 50] but their biological implication to the MSC lineage remains poorly known. Furthermore, *in vitro* EGF treatment was enough to reduce the expression of both cell markers, confirming an osteogenic role by EGF on DPSCs. The cell differentiation trigger changes in the immunophenotype of DPSCs, a test that can be used to monitor cell differentiation. We have found that there is a strong relationship between CD146 and CD10 expression levels and the osteogenic differentiation of DPSCs because these markers are related with the stemness of these cells. After 7 days, we observed stronger surface marker suppression with EGF but it is clear that this criterion is not enough to consider it as osteogenic differentiation; however, it can be useful to follow the DPSC-osteoblast transition process. Nonetheless, it would be necessary to enlarge this kind of assays to characterize the behavior of other surface markers associated with the stemness of MSCs. Additionally, osteogenic *in vitro* differentiation of MSCs is commonly evidenced by early ALP activity, extracellular matrix mineralization and expression of typical osteoblast markers [51–53]. In agreement with our experiments, an increase of mRNA expression of ALP was observed in cells cultured with EGF. In addition, it is well known that OCN is an important osteogenic marker which regulates the formation of mineralization nodules and hence, leads to osteogenesis [54]. In this context, the upregulation of OCN expression as results from EGF treatment strengthen this study, suggesting its osteogenic effect. OPN, another important marker of late-stage osteoblast differentiation [55], was also overexpressed when cells were cultured with EGF, confirming its osteogenic role.

To our knowledge, this is the first report that evaluates the osteogenic effects of EGF on DPSCs; however, to elucidate the mechanism by which this occurs as well as its efficacy in animal models, further studies are required.

In conclusion, this study demonstrates that EGF plays an enhancer role on osteogenic differentiation of DPSCs because it is capable of increasing extracellular matrix mineralization. A low concentration of EGF (10 ng/ml) is sufficient to induce morphological and phenotypic changes; however, bFGF at an equal concentration exerts an inhibitory effect. These data suggests that DPSCs in combination with EGF could be an effective stem cell-based therapy to bone tissue engineering applications in periodontics and oral implantology.
